# Reduction of vertebral height with fragility vertebral fractures can induce variety of neurological deterioration

**DOI:** 10.1186/s13018-017-0649-1

**Published:** 2017-10-03

**Authors:** Kazuhiro Fujimoto, Tsukasa Kanchiku, Yasuaki Imajo, Hidenori Suzuki, Norihiro Nishida, Masahiro Funaba, Toshihiko Taguchi

**Affiliations:** Department of Orthopaedic Surgery, Yamaguchi University Graduate School of Medicine, 1-1-1 Minami Kogushi, Ube, Yamaguchi 755-8505 Japan

**Keywords:** Spinal fractures, Osteoporotic fractures, Thoracic vertebrae, Lumbar vertebrae, Conus medullaris, Spinal cord compression, Neurological examination

## Abstract

**Background:**

The presence of vertebral fractures affect variations in the termination level of conus medullaris (TLCM) and alter neurological findings. However, few studies have examined association between vertebral fractures, TLCM, and neurological findings. Thus, we herein studied the number and severity of vertebral fractures, TLCM, and neurological findings to clarify the mechanism of neurological deterioration in patients with vertebral fractures.

**Methods:**

A total of 411 patients who underwent computed tomographic myelography were classified into those with (group F, *n* = 73) and those without vertebral fractures (group C, *n* = 338). We assessed correlations between TLCM and age, height, and gender in group C, differences in TLCM between groups F and C, and correlations between TLCM, and the number and severity score of fractures. Neurological evaluations were performed for the patellar tendon reflex (PTR), muscle weakness, sensory disturbance, and bladder contraction disorders.

**Results:**

TLCM was most commonly located at the L1 vertebral body in group C and did not significantly differ with age, height, or gender. TLCM was most commonly located at L2 vertebral body in group F. TLCM was more caudally located in group F (*P* < 0.01). Additionally, there was a significant difference between TLCM and number of fractures, and the severity score of fractures (both *P* < 0.01). Twenty-three patients showed neurological deterioration by vertebral fractures. Some patients with T12 vertebral fracture showed hyperreflexia of PTR. Serious bladder contraction disorders were seen in patients with compression at close range of TLCM.

**Conclusion:**

We confirmed that vertebral fractures altered location of the TLCM, thus altering potential neurological symptoms. Moreover, there were correlations of the TLCM with the number and severity score of vertebral fractures. Spine surgeons should be cognizant of the relationship between TLCM, level of compressive lesion, and neurological findings to avoid the wrong level in spine surgery and unexpected neurological deteriorations after surgery.

## Background

Variations in the termination level of the conus medullaris (TLCM) range from the lower third of T11 vertebral body to the upper third of L3 vertebral body [1, 2]. Specifically, several studies have reported that the TLCM was located mainly at the L1 vertebral body, as visualized by magnetic resonance imaging (MRI) in normal living subjects [1–3].

The number of patients with osteoporotic vertebral fractures is increasing because of the progressive aging of society as well as increased availability of diagnostic modalities [4, 5]. In comparison to subjects without vertebral fractures, the presence of vertebral fractures affect variations in the TLCM and alter neurological findings because of shortening of the total spinal length due to vertebral fractures with no change in the length of the spinal cord. We believe the presence of more severe and more numerous vertebral fractures affect variations in the TLCM and alter neurological findings. However, few studies have examined association between vertebral fractures, TLCM, and neurological findings. Thus, we herein studied the number and severity of vertebral fractures, TLCM, and neurological findings to clarify the mechanism of neurological deterioration in Japanese patients with fragility vertebral fractures.

## Methods

### Patients

**Fig. 1:**
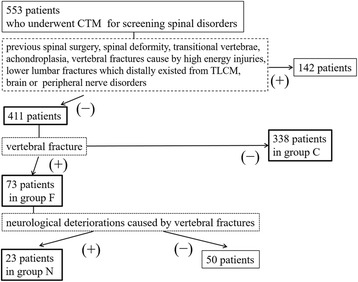
Study flowchart including subject recruitment

Patients who underwent whole spine computed tomographic myelography (CTM) for screening of spinal disorders between January 2012 and December 2014 were recruited for this study. We excluded patients with previous spinal surgery, spinal deformity, transitional vertebrae, achondroplasia, vertebral fractures caused by high energy injuries, or brain or peripheral nerve disorders. We also excluded the lower lumbar fractures which caudally existed from TLCM because they cause the spinal cord redundant. Ultimately, a total of 411 patients were enrolled in this study, as shown in Fig. 1. We grouped patients into those with vertebral fractures (group F, *n* = 73) and those without vertebral fracture (group C, *n* = 338). In group F, there were 22 males (30%) and 51 females (70%), with mean age of 68 ± 14 years (range, 22–84 years) and mean height of 154.0 ± 9.3 cm (range, 134–178 cm). In group C, there were 194 males (57%) and 144 females (43%), with mean age of 64 ± 14 years (range, 20–95 years) and mean height of 159.6 ± 9.4 cm (range, 131–184 cm). All study participants provided informed consent, and the study design was approved by the institutional review board of Yamaguchi University Hospital (control number of H26-100).

### CTM testing

**Fig. 2:**
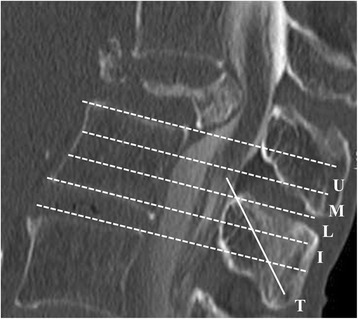
Termination level of the conus medullaris. The termination level of the conus medullaris (T) was decided into four levels: the intervertebral disc (I) and the three equal parts [upper (U), middle (M), and lower (L) third] of the vertebral body

The TLCM was assessed using whole spine CTM. All patients were evaluated by computed tomography with either SOMATOM Sensation 64® or SOMATOM Definition® (both from Siemens Healthcare, Erlangen, Germany), with a slice thickness of 1 mm and intersection gap of 1 mm for sagittal sequence. The TLCM was classified into four levels, with the vertebral body divided into three equal portions (upper, middle, and lower third) and the intervertebral disc defined as a separate level (Fig. 2).

### Assessment of TLCM and vertebral fractures

First, the correlation between the TLCM and gender, age, and height in group C was assessed. Second, differences in the TLCM between groups F and C were evaluated. In group F, the number of vertebral fractures were determined, and the severity of vertebral fractures was graded using a semiquantitative method, shown in Table 1 [6]. Three specialists in spine surgery independently graded the TLCM and fractures in all patients, and they were blinded of the patient’s age, gender, and height. The relationships of the TLCM and number and severity score of vertebral fractures were determined.

**Table 1:** Severity score of vertebral fractures

Grade (point)	Reduction rate (%)	
	Height	Area
Mild (1)	20–25	10–20
Moderate (1.5)	25–40	20–40
Severe (2)	Over 40	Over 40

Vertebral fractures were graded using a semiquantitative method as mildly (1 point), moderately (1.5 point), and severely (2 points) deformed. Mildly deformed was defined as approximately 20–25% reduction in anterior, middle, and/or posterior height and a reduction of area 10–20%, moderately deformed as approximately 25–40% reduction in any height and a reduction of area 20–40%, and severely deformed as approximately over 40% reduction in any height and a reduction of area over 40%. The severity score was defined as total point of vertebral fractures

### Assessment of neurological findings

In group F, 23 of a total of 73 patients showed muscle weakness and/or sensory disturbances, and/or bladder contraction disorders due to vertebral fractures at the level of the rostral conus medullaris, and were subcategorize to group N (Fig. 1). Neurologic evaluations were performed for the patellar tendon reflex (PTR), muscle weakness, sensory disturbance, and bladder contraction disorders. PTR was classified as hyperreflexia, normal reflex, hyporeflexia, or areflexia. Muscle weakness was evaluated by manual muscle testing (MMT). We defined muscle weakness as MMT ≤ 4. Sensory disturbance was evaluated by the pin-prick test using Brain and Walton’s dermatome [7]. Bladder contraction disorder was classified as severe (urinary retention and/or incontinence), moderate (sense of retention and/or dribbling and/or thin steam and/or incomplete continence), and mild (urinary retardation and/or pollakiuria). Compressive lesions of the spinal cord were also assessed using CTM and classified into five groups: vertebral body level of T10 (*n* = 2), T11 (*n* = 3), T12 (*n* = 14), L1 (*n* = 3), and L2 (*n* = 1). The correlation between neurological findings and the TLCM was also determined.

### Statistical analysis

The Mann-Whitney *U* and Kruskal-Wallis tests were used for unpaired comparisons of patient data. The correlation between the TLCM and severity score of vertebral fractures with group N and correlations between the TLCM and age and height in group C were assessed using Spearman’s rank correlation coefficient. *P* values of less than 0.05 were considered statistically significant. For all statistical analysis, JMP® 11 (SAS Institute, Cary, NC, USA) was used.

## Results

**Fig. 3:**
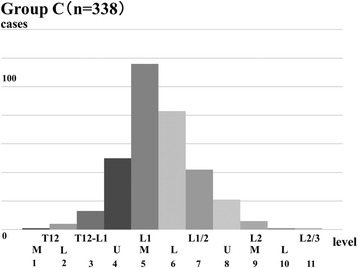
Range of the termination level of the conus medullaris in group C. The range of the termination level of the conus medullaris extended from the lower third of the T12 to the L2–3 intervertebral body level in group C; it was most commonly located at the L1 vertebral body level (74%). The *X*-axis shows the numbers corresponding to the termination level of the conus medullaris, such as (1) for T12-M and (2) for T12-U

The TLCM ranged from the middle third of the T12 vertebral body to the L2–3 intervertebral disc in group C; the TLCM was most commonly located at the L1 vertebral body (249/338, 74%; Fig. 3). The TLCM did not significantly correlate with age (rs = 0.0617, *P* = 0.26), or height (rs = − 0.0087, *P* = 0.87). There was no significant difference between the TLCM and gender (*P* = 0.74) in group C.

**Fig. 4:**
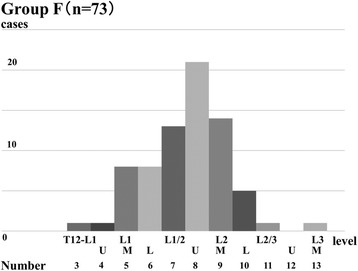
Range of the termination level of the conus medullaris in group F. The range of the termination level of the conus medullaris extended from the lower third of the T12–L1 intervertebral body to the L3 vertebral body level in group F; the conus was most commonly located at distal L2 vertebral body level (42%)

In contrast, the TLCM ranged from the T12 to L1 intervertebral disc to the middle third of the L3 vertebral body in group F; the TLCM was most commonly located at the distal L2 vertebral body (31/73, 42%; Fig. 4). There was a significant difference in the TLCM between groups C and F (*P* < 0.01).

**Fig. 5:**
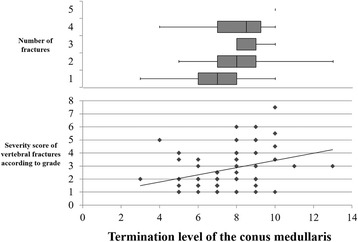
Termination level of the conus medullaris and number of vertebral fractures, severity score of vertebral fractures. The mean termination level of the conus medullaris significantly related to the number of fractures (*P* < 0.01). Correlation between the termination level of the conus medullaris and the severity score of vertebral fractures was also seen significantly (*P* < 0.01)

The locations of vertebral fractures were T6 1 case, T7 6, T8 10, T9 6, T10 8, T11 19, T12 34, L1 29, and L2 15. In addition, there was a significant difference between the TLCM and the number of vertebral fractures (*P* < 0.01, Fig. 5). Furthermore, the TLCM significantly correlated with the severity score of vertebral fractures (rs = 0.3599, *P* < 0.01, Fig. 5).

Neurological deteriorations in group F were shown in Table 2. Muscle weakness was shown the most proximal denervation muscle.

**Table 2:** Summary of patient characteristics in group N

Case	Gender	Age	Level of fracture	TLCM	PTR	Muscle weakness	Sensory disturbance	Bladder contraction disorders
1	Female	80	T10	L2U	↑	IP	L1	N
2	Female	72	T10	L1M	↑	IP	L2	N
3	Female	75	T11	L2U	↑	IP	L3	Mild
4	Female	80	T11	L1L	−	IP	L4	Mild
5	Male	71	T11	T12/L1	↓	QC	L4	N
6	Male	81	T12	L2M	↑	IP	L5	N
7	Female	79	T12	L2U	↑	IP	L5	Mild
8	Female	72	T12	L2U	↑	N	L5	N
9	Female	62	T12	L2U	↓	N	S1	Mild
10	Female	71	T12	L2U	↓	IP	L4	Mild
11	Female	72	T12	L2U	↓	QC	L4	N
12	Male	78	T12	L2U	↓	IP	L1	N
13	Female	71	T12	L1/2	−	N	L4	Mild
14	Female	76	T12	L1/2	−	TA	L5	Severe
15	Female	84	T12	L1/2	→	IP	L4	Mild
16	Female	71	T12	L1L	↓	N	L5	Moderate
17	Male	75	T12	L1L	↓	TA	L5	Severe
18	Female	63	T12	L1M	↓	TA	N	N
19	Male	75	T12	L1M	→	TA	N	Moderate
20	Female	65	L1	L2M	→	N	S1	Moderate
21	Female	22	L1	L2U	→	TA	L5	Moderate
22	Female	63	L1	L2U	→	TA	L4	N
23	Female	39	L2	L2/3	→	IP	L2	N

*TLCM* termination level of the conus medullaris, *U* upper third, *M* middle third, *L* lower third, *PTR* patellar tendon reflex, ↑ hyperreflexia, → normalreflexia, ↓ hyporeflexia, − areflexia, *IP* iliopsoas, *QC* quadriceps, *TA* tibialis anterior, *N* normal findings

## Discussion

Generally, location of the TLCM defines neurological symptoms. In the present study, we confirmed that vertebral fractures altered location of the TLCM, thus altering potential neurological symptoms. In each patient with vertebral fractures and who exhibit neurological deterioration, the TLCM and neurological findings differed in comparison to subjects without vertebral fractures. One mechanism to explain our findings is shortening of the total spinal length due to vertebral fractures with no change in the length of the spinal cord. To the best of our knowledge, this is the first report describing correlations of the TLCM with the number and severity score of vertebral fractures.

Our results of group C cases concur with several previous studies showed by MRI that the TLCM was mainly located at the L1 vertebral body in normal living subjects [1–3]. Some reports were described that TLCM was more caudally located in females [1, 8, 9] and the elderly [1] and showed differences between races [10–12]. In addition, previous reports revealed that the presence of spinal deformities and lumbosacral transitional vertebrae were associated with variations in the TLCM [13–16]. However, the TLCM was not affected by age, height, or gender in this cohort of Japanese subjects. Moreover, we believed that assessing of the TLCM using only MRI is not enough to assess the transitional vertebrae and spinal deformity. Importantly, our results were confirmed by the assessment of the transitional vertebrae and spinal deformity using of the whole spine CTM.

We recently reported that the L4 segment of the spinal cord was present from the middle third of the T11 vertebral body to the T11–12 intervertebral disc level based on neurological findings in patients with a single ossification of ligamentum flavum and without vertebral fracture [17]. Generally, hyperreflexia of PTR is observed in patients with upper neuron disorders; nevertheless, non-hyperreflexia of the Achilles tendon reflex is sometimes observed in elderly patients with upper neuron disorders [18–20]. Patients with T12, or lumbar vertebral body fractures, are not supposed to exhibit hyperreflexia of PTR as the reflex center of PTR is the L4 segment of the spinal cord [21]. However, in the present study, 3 of 4 patients with T12 vertebral fractures showed hyperreflexia of PTR and the TLCM located at the L2 vertebral body distally in every three cases. These findings suggested a disorder of the L3 or more rostral segment of the spinal cord occurred at the T12 vertebral body level. Serious bladder contraction disorders were seen in patients with compression at close range of TLCM; however, mild bladder contraction disorders were seen in patients with T11, T12, or L1 vertebral fractures. Previous report revealed the L4–S1 segments of the spinal cord occurred approximately 1.6 vertebral body superior from the TLCM [22] and the S2–5 segments of spinal cord occurred between 0 and 1.0 vertebral body superior from the TLCM [23] in patients with and without vertebral fractures. Our results of neurological findings concur with these previous reports. Clinicians should pay attention to the TLCM and neurological findings when the presence of vertebral fractures or caudal TLCM is revealed by MRI or CTM. Vertebral fractures alter location of the TLCM, thus altering potential neurological symptoms. The wrong level in spine surgery and unexpected neurological deteriorations after surgery are the complications associated with diagnosing neurological symptoms incorrectly. To avoid these complications, spine surgeons should compensate for the movement of the TLCM when the patients with vertebral fracture show atypically neurological findings at each disordered level. For farther works, we will investigate of the frequency of clinically significant myelopathy after fragility vertebral fractures and of the potential interventions aimed at preventing reduction of vertebral height and myelopathy to clarify the mechanism of neurological deterioration in patients with fragility vertebral fractures.

Moreover, biomechanical study revealed that the compressive lesions at the thoracolumbar junction showed spinal cord segment disorders rather than nerve root disorders because nerve roots exhibited higher strength than the gray and white matters of the spinal cord [24]. However, clinicians never forget that vertebral fractures at the thoracolumbar junction sometimes cause nerve root disorders, as shown in the case 23. The symptoms may be associated with the strength characteristics of these tissues, in addition to the anatomical complexity and the manner of compression [25, 26].

The limitations of the present study included the small number of patients, particularly in group N, and the wide variety of diseases in group C, such as compressive cervical myelopathy, compressive thoracic myelopathy, lumbar spinal canal stenosis, and low back pain. However, our findings also showed the usefulness of diagnosing patients with vertebral fractures. Clinicians should pay particular attention to the presence of vertebral fractures as the TLCM and neurological findings slightly differ between patients with and without vertebral fractures.

## Conclusion

We found that vertebral fractures altered location of the TLCM, thus altering potential neurological symptoms. Moreover, there were correlations of the TLCM with the number and severity score of vertebral fractures. Spine surgeons should be cognizant of the relationship between TLCM, level of compressive lesions, and neurological findings to avoid the wrong level in spine surgery and unexpected neurological deteriorations after surgery.

## Abbreviations

CTM: Computed tomographic myelography
MMT: Manual muscle testing
MRI: Magnetic resonance imaging
PTR: Patellar tendon reflex
TLCM: Termination level of the conus medullaris
